# Can Infinitival *to* Omissions and Provisions Be Primed? An Experimental Investigation Into the Role of Constructional Competition in Infinitival *to* Omission Errors

**DOI:** 10.1111/cogs.12407

**Published:** 2016-10-20

**Authors:** Minna Kirjavainen, Elena V. M. Lieven, Anna L. Theakston

**Affiliations:** ^1^ School of Psychological Sciences University of Manchester; ^2^ Foreign Language Department Osaka Gakuin University

**Keywords:** Structural priming, Implicit learning, Constructional competition, To‐infinitive, Grammatical errors, First language acquisition

## Abstract

An experimental study was conducted on children aged 2;6–3;0 and 3;6–4;0 investigating the priming effect of two WANT‐constructions to establish whether constructional competition contributes to English‐speaking children's infinitival *to* omission errors (e.g., *I want ___ jump now). In two between‐participant groups, children either just heard or heard and repeated WANT‐to, WANT‐X, and control prime sentences after which to‐infinitival constructions were elicited. We found that both age groups were primed, but in different ways. In the 2;6–3;0 year olds, WANT‐to primes facilitated the provision of *to* in target utterances relative to the control contexts, but no significant effect was found for WANT‐X primes. In the 3;6–4;0 year olds, both WANT‐to and WANT‐X primes showed a priming effect, namely WANT‐to primes facilitated and WANT‐X primes inhibited provision of *to*. We argue that these effects reflect developmental differences in the level of proficiency in and preference for the two constructions, and they are broadly consistent with “priming as implicit learning” accounts. The current study shows that (a) children as young as 2;6–3;0 years of age can be primed when they have only heard (not repeated) particular constructions, (b) children are acquiring at least two constructions for the matrix verb WANT, and (c) that these two WANT‐constructions compete for production.

## 1 Introduction

Children's grammatical errors are a widely investigated phenomenon in child language research because they provide clues as to what the building blocks of children's developing language are and what kind of strategies children rely on when producing/comprehending language. One such error pattern is children's infinitival *to* omissions (Bloom, Tackeff, & Lahey, 1984; Diessel, 2004; Kirjavainen & Theakston, 2011; Kirjavainen, Theakston, Lieven, & Tomasello, 2009; Landau & Thornton, 2011; Limber, 1973; Pinker, 1984). Infinitival *to* is an obligatory marker of to‐infinitive constructions such as in example (1). However, 2–3‐year‐old English‐speaking children commonly omit this marker and produce erroneous utterances such as in (2).

1. I want to hold Postman Pat
*I want hold Postman Pat

In this article, we investigate whether these errors can be explained by the usage‐based/constructivist account of first language acquisition (e.g., Goldberg, 2006; Tomasello, 2003). More specifically, we investigate whether constructional competition (Kirjavainen & Theakston, 2011; Kirjavainen et al., 2009) contributes to the observed error pattern.

The usage‐based/constructivist approach assumes no pre‐given linguistic knowledge. Instead, it holds that a central part of children's language acquisition is the memorization of words and multiword constructions. Children both break down and expand those stored sequences to make abstractions, a process that, over time, results in the acquisition of grammar. This is a gradual process, and it reflects the distributional properties of the input (see, e.g., Ambridge, Kidd, Rowland, & Theakston, 2015, for an overview), namely high‐frequency constructions, and constructions that exhibit greater variability in input (and output) are likely to be learned earliest, whereas lower frequency constructions will be learned later and are more prone to error. This view differs from structurally oriented and universal grammar approaches to language acquisition, which posit innate grammatical categories and rule application, and assume early abstraction of grammatical information (e.g., Pinker, 1984). As our aim is to investigate the constructivist explanation of infinitival *to* omissions, we will not here consider in detail alternative explanations for these errors, or focus on testing their predictions. For information on alternative accounts of infinitival *to* omissions, see, for instance, Landau and Thornton (2011), and for empirical tests of related predictions, see, for instance, Kirjavainen et al. (2009).

In the current paper, we use a syntactic priming paradigm to assess whether infinitival *to* omission/provision in the WANT‐to‐V construction is affected by exposure to and/or repetition in the prior discourse of (a) the WANT‐to (e.g., *I* *want to* *ride my bike*) construction, which is the overwhelmingly most common (42%) WANT + word combination in British child‐directed speech (Manchester corpus, Theakston, Lieven, Pine, & Rowland, 2001) and (b) the more abstract WANT‐X construction (e.g., *I want my bike*) in which considerable lexical variability is found (in the X slot).^1^

We will first review previous research on infinitival *to* errors conducted within a constructivist framework and explain the rationale for the current study. We will then review a number of priming studies that are relevant to the present investigation. Lastly, we will present our current study and discuss the results in the context of “priming as implicit learning” accounts (e.g., Chang, Dell, & Bock, 2006).

### 1.1 Infinitival to omissions

It is well‐documented that 2‐ and 3‐year‐old English‐speaking children produce errors in which they omit infinitival *to* in obligatory contexts (e.g., Bloom et al., 1984; Diessel, 2004; Kirjavainen et al., 2009; Limber, 1973). These omissions cannot be explained by any obvious developmental factors such as (a) performance limitations whereby words carrying less semantic information (i.e., *to*) are omitted in children's language due to their limited processing power (e.g., Bloom, 1990; Valian & Aubry, 2005; Valian, Hoeffner, & Aubry, 1996) because to‐infinitival utterances with and erroneously without *to* do not differ in their mean length of utterance (Kirjavainen et al., 2009)^2^ or (b) a lack of lexical or at least rudimentary constructional knowledge, since the provision and omission of *to* in to‐infinitival utterances co‐occur in children's speech, even within utterances that share the same matrix (i.e., main clause) verb (Kirjavainen et al., 2009). The latter indicates that children have at least some knowledge of the fact that certain matrix verbs occur in two (or more) sentence types (those in which *to* is produced and those in which it is not), but for some reason fail to provide *to* in obligatory contexts consistently. Also, the suggestion whereby an initial perceptual problem in detecting infinitival *to* in language input results in children assuming early in development that some matrix verbs can appear with or without *to* (Pinker, 1984) struggles to explain the behavior of, at least, some children. Kirjavainen et al. (2009) found that infinitival *to* omission and provision tended to emerge at the same developmental point in a densely collected corpus from one English‐speaking child, Thomas (Lieven, Salomo, & Tomasello, 2009) or, in the case of some verbs, correct productions preceded omissions by several months. In addition, Thomas had been producing prepositional *to*, assumed to be the key lexical item in the recovery from infinitival *to* omissions (Pinker, 1984), for several months before the first infinitival *to* omission was found in his corpus.

### 1.2 A constructivist explanation for infinitival to omissions

Within a constructivist framework, children are thought to be learning a number of sentence constructions directly from the language that they hear. These constructions can be specified at a number of levels, from the fully lexically specified (e.g., *I want to go*), to the more abstract (e.g., *NP‐VP‐to‐VP*). The precise level of representation is assumed to reflect the distributional characteristics of the input, such that high token frequency of a specific exemplar promotes the acquisition of lexically specific constructions, whereas greater variability results in abstraction and generalization (Bybee, 1995). In the case of infinitival constructions, the input to young children is characterized by the repeated occurrence of a small number of matrix verbs (e.g., *want*,* go*,* need*) combined with a larger number of complement verbs. Thus, the most likely constructions to emerge in children's speech are partially lexically specified (e.g., *I want to Verb*,* I'm going to Verb*). However, many of these matrix verbs also appear in other kinds of sentence constructions, for example the simple transitive (*I want a drink*) and the simple intransitive (*I'm going to the shops*), as well as other more complex infinitival constructions (e.g., *I want Mummy to read a story*). To the extent that these same matrix verbs appear in other constructions in the input frequently, and with a range of different complements, children are expected to also derive alternative partially lexically specified constructions such as WANT‐X and *going*‐X. The scope of “X” in these constructions will be determined by the variability in the items appearing in that slot in the input, their semantic heterogeneity, and their individual frequencies. To further complicate matters, some matrix verbs can also be used in contracted forms such as *wanna* and *gonna*.

Constructions are paired form‐meaning mappings, and therefore when children learn different kinds of constructions, they are also learning to associate these with particular meanings. In instances where there is lexical overlap between constructions, for example between WANT‐to and WANT‐X, there is also likely to be some degree of overlap in their perceived meanings. This leads to the possibility of competition between constructions, especially where there is some degree of overlap in meaning, and forms the basis for the constructivist account of infinitival *to* omission errors.

Kirjavainen and colleagues (2009, 2011) investigated infinitival *to* omission errors in WANT‐to‐V and *going*‐to‐V constructions from the constructivist/usage‐based viewpoint. Their (2009) study suggested that competition between the more abstract verb‐X (e.g., WANT‐X, *I want juice/apple/my teddy*) and the more lexically specific verb‐to (e.g., WANT‐to, *I want to eat it/hold teddy/go under the table*) constructions may be one reason for children's infinitival *to* omission errors. More specifically, they argued that children hear matrix verbs that occur in infinitival *to* and V‐NP constructions resulting in children building at least two representations of those matrix verbs, one with and one without *to*. Following Bybee (1998), they hypothesized that the relatively high token frequency of the WANT‐to and *going*‐to constructions in the input addressed to children would result in lexically specific WANT‐to(‐V) and *going*‐to(‐V) constructions being acquired and produced with a variety of verb complements. At the same time, the high type frequency in the input of the WANT‐X and *going*‐X constructions (in which X denotes any other word than *to* following WANT/*going*, no combination of which occurred in the input with particularly high individual token frequency) would result in another, more abstract, representation being associated with these matrix verbs. In this more abstract representation, a number of different lexical items can follow the verb. Note that this approach does not imply that any word can be used in the X‐slot. As modeled by the input, it is likely that the words that X stands for are articles, (pro)nouns, adjectives, and (for *going*) prepositions; thus, the WANT‐X construction includes utterances predominantly of the following types: WANT‐NP and WANT‐NP‐to‐VP. However, there can be semantic overlap between associated constructions, for example the implication of action/possession in many WANT‐X as well as WANT‐to‐VP utterances, or of movement in many *going‐*X as well as *going*‐to‐VP utterances. Coupled with the observations that (a) infinitival *to* lacks perceptual salience (Pinker, 1984), and (b) in English, many nouns can also be used as verbs (e.g., *a brush/to brush*), Kirjavainen et al. (2009) argued that these properties of the language may lead children to assume that verbs are also acceptable fillers for the “X” slot, accounting in part for infinitival *to* omission errors.

Note, however, that it is important to consider the direction of these hypothesized effects. For example, the substitution of verbs into the X‐slot of the WANT‐X construction is motivated by semantic similarities between the action or possession‐like meanings of the WANT‐to and WANT‐X constructions, the noun/verb overlap, and the lack of perceptual salience of infinitival *to*. In contrast, the properties of the VP‐slot in the WANT‐*to*‐VP construction are likely to be more tightly specified as this construction only occurs with verbs. Thus, although children may incorrectly produce nouns in this construction, this is likely to be relatively infrequent (Kirjavainen et al., 2009).

Following MacWhinney and colleagues (e.g., Bates & MacWhinney, 1987; MacWhinney, 1987), Kirjavainen et al. (2009) assumed that related constructions compete when children are selecting utterance types for production (see also Ambridge, Pine, & Rowland, 2012; Theakston & Lieven, 2008, for competition effects between other sentence types). Because both WANT/*going‐*X and WANT/*going‐to* constructions are hypothesized to be (at least partially) compatible with the semantics associated with infinitival contexts at this stage in development, which of the two identified constructions for each matrix verb wins out, leading to either provision or omission of infinitival *to* in obligatory contexts, was assumed to reflect the relative frequency of these constructions in child‐directed speech. To test this hypothesis, Kirjavainen et al. (2009) studied 13 children's longitudinal corpora between the ages of approx. 2;0–3;0 and calculated infinitival *to* omission error rates and input frequencies for different construction types for WANT and *going*. They found that, in terms of proportional use, the children who had more WANT‐to and *going*‐to input relative to WANT‐X and *going‐X* input produced fewer infinitival *to* omissions than children whose input contained more instances of the WANT/*going*‐X constructions. Moreover, the verb (WANT vs. *going*) which was proportionally more frequent in the input in the VERB‐X construction was the verb with which the children made more infinitival *to* omission errors.

Kirjavainen and Theakston (2011) investigated the constructional competition account for infinitival *to* omissions further. They conducted a corpus study on the same 13 children as Kirjavainen et al. (2009) but instead of looking at the effect of input in the children's language exposure overall, they investigated the role of the immediate discourse context (previous 10 utterances) on the production of infinitival *to* omissions with the most common infinitival *to* matrix verb, WANT. They reasoned that if children are learning two constructions for WANT,^3^ the immediate discourse context should differentially affect the provision of infinitival *to* in obligatory contexts. More specifically, it should be possible to find a priming effect such that the children who had recently heard or themselves produced at least one instance of the supporting WANT‐to construction should provide *to* in obligatory contexts relatively more frequently than if they had heard or produced the competing WANT‐X construction, or if there were no relevant primes. On the other hand, infinitival *to* omissions should be produced relatively more often following instances of the WANT‐X construction than after instances of the WANT‐to construction, or if there were no relevant primes. This is largely what was found. The children's infinitival *to* omissions/provisions were primed by their own utterances in the previous discourse. Their interlocutors' discourse utterances also had an effect on the provision/omission of *to* but, due to differences in the types of WANT‐constructions children and parents produce, the interlocutors' priming effect was weaker than the effect found from self‐priming. While young children's WANT‐utterances predominantly instantiate two types of egocentric constructions, namely *I‐want‐X* or *I‐want‐(to)‐V*, caregivers' WANT‐utterances are more varied (e.g., *I‐want‐X*,* I‐want‐to‐Verb*,* I‐want‐NP‐to‐V*,* NP‐wants‐NP‐to‐V*) and commonly focus on joint needs and actions (*I‐want‐you‐to‐V*,* Do‐you‐want‐me‐to‐V?*) rather than their own. These discourse factors result in greater lexical and structural specificity and overlap between the children's target utterances and their own prior utterances than those produced by their caregiver. Together these studies suggest that (a) children are learning two constructions (VERB‐to and VERB‐X) for matrix verbs taking to‐infinitive complements, and (b) both the relative input frequencies and input/output in the immediate discourse context preceding a child's production of a to‐infinitive construction affect the child's infinitival *to* omissions.

However, although Kirjavainen et al.'s (2009) previous studies provide support for a constructivist account of infinitival *to* omission errors, this support is solely based on corpus analyses of naturalistic data. One issue with corpus‐based studies is that it is difficult to control important distributional properties of the language that may affect the linguistic feature under investigation, for instance (a) the number of times a given construction (e.g., WANT‐X) occurs in the prior discourse, (b) the number of times a given word occurs in discourse overall (e.g., the matrix verb WANT, prepositional *to*, or infinitival *to* with other matrix verbs), or (c) the frequencies of different lexical items (e.g., complement verbs) in a variety of different constructions in the child's language and input overall. In the current study, we address these issues head‐on by conducting an experimental investigation using a syntactic priming paradigm in which we can better control many of these factors. Before we describe the current study, a short overview of the relevant priming literature is given.

### 1.3 Priming in children

*Contextual persistence* or *priming* is a phenomenon whereby speakers are likely to produce the same syntactic construction or word they have just been exposed to or have themselves just uttered than an alternative construction/word to convey the same meaning. For example, when describing a transitive scene, a person who has heard/produced a passive sentence is more likely to go on to produce a(nother) passive sentence to convey the transitive meaning relative to contexts when they have previously heard/produced an active sentence. A large number of studies have demonstrated this effect for several linguistic construction types in adults and in children both in naturalistic conversational (De Marneffe, Grimm, Arnon, Kirby, & Bresnan, 2012; Gries, 2005; Kirjavainen & Theakston, 2011; Pickering & Garrod, 2004; Theakston & Lieven, 2008; see also Bloom, Rispoli, Gartner, & Hafitz, 1989) and experimental contexts (e.g., Bencini & Valian, 2008; Bock, 1986; Bock, Dell, Chang, & Onishi, 2007; Bock & Loebell, 1990; Branigan, Pickering, & Cleland, 2000; Branigan, Pickering, Liversedge, Stewart, & Urbach, 1995; Chang et al., 2006; Ferreira, 2003; Huttenlocher, Vasilyeva, & Shimpi, 2004; Messenger, Branigan, & McLean, 2011; Pickering & Branigan, 1998; Potter & Lombardi, 1998; Rowland, Chang, Ambridge, Pine, & Lieven, 2012; Savage, Lieven, Theakston, & Tomasello, 2003, 2006; Shimpi, Gamez, Huttenlocher, & Vasilyeva, 2007; Vasilyeva & Waterfall, 2012; Whitehurst, Ironsmith, & Goldfein, 1974).

The effect is assumed to result from the discourse utterance either (a) causing a short‐term activation of its syntactic structure and of the lexical items encountered in it (e.g., Branigan et al., 2000; Pickering & Branigan, 1998), (b) strengthening an existing syntactic representation (i.e., priming reflects a process of implicit learning) (e.g., Bock & Griffin, 2000; Chang et al., 2006), or (c) activation of a discourse function associated with a given prime sentence (Vasilyeva & Waterfall, 2012). The second of these accounts, priming as implicit learning, has particular relevance for the process of language acquisition. Here, priming is viewed as a form of language input, which can serve the same functions as the other input children experience in terms of shaping their linguistic representations, and driving the process of abstraction. According to the implicit learning account, priming effects are strongest when the sentence construction encountered fails to match with that which the listener expects to occur. Expectation should reflect the distributional properties of the language, such that, all else being equal, lower frequency constructions will be more surprising and thus exert a greater priming effect. However, during language acquisition, the child's weaker sentence representations are thought to be altered and strengthened more by encountering new instances than the relatively stable representations of adult speakers (e.g., Rowland et al., 2012; Savage et al., 2006; and see Ambridge et al., 2015, for a review of frequency effects in acquisition). In cases such as infinitival *to* omissions, in which the child's representations are non‐adult‐like and must necessarily change over development, and in which priming between different constructions (i.e., WANT‐NP priming WANT‐V construction) might take place, the question of whether and how priming might operate is of theoretical significance.

Regardless of the fact that there is disagreement as to what process underlies priming, it is generally agreed that priming is caused by an *existing representation(s)* being activated/strengthened. This means that if children have already acquired at least some knowledge of two constructions with matrix verbs such as WANT (e.g., WANT‐to and WANT‐X), and if competition between these two constructions contributes to infinitival *to* omission errors, we should be able to observe a priming effect whereby exposure to the WANT‐to construction increases the provision of infinitival *to* in obligatory contexts, whereas exposure to the WANT‐X construction increases the production of infinitival *to* omission errors (i.e., WANT‐X inhibits the provision of *to*).

The implicit learning account assumes that constructions that are not preferred for a given discourse context should show stronger priming effects than those that are preferred. In the context of development, and especially in cases where children are observed to produce grammatical errors, this assumption leads to some interesting predictions. Previous corpus evidence shows that on average 58% of input directed to children containing the verb WANT takes the form WANT‐X (Kirjavainen et al., 2009), and the WANT‐X construction is acquired earlier than the WANT‐to construction (Kirjavainen et al., 2009). We might therefore predict that young (<3;0) children will have stronger representations of WANT‐X than WANT‐*to*, and thus prefer to use the WANT‐X construction over the WANT‐*to* construction. Consequently, in line with implicit learning accounts, children in our younger age group might show stronger priming effect for the lesser known WANT‐*to* primes (weaker representation) than WANT‐X primes (preferred representation). However, as children move toward a more adult‐like form‐function mapping for the to‐infinitive construction, the relative weighting of the two constructions is expected to shift, resulting in changes in the priming effects observed.

#### 1.3.1 Age and conditions under which children can be primed

Previous studies indicate that priming effects can be found for children even before 3 years of age (Kirjavainen & Theakston, 2011; Shimpi et al., 2007; Theakston & Lieven, 2008) and that children can show relatively strong priming effects when they are 3 and 4 years of age (Rowland et al., 2012). Thus, it is not unreasonable to assume that those types of constructions that children learn early in development (e.g., I‐WANT‐X and I‐WANT‐*to*‐VP), may be available for priming in experimental contexts during the latter half of the third year of life (2;6–3;0) regardless of whether the children's representations are yet adult‐like.

It seems to be the case, however, that in very young children (aged <3 years) strong priming effects can be obtained only if the children have themselves produced or (in experimental contexts) repeated the prime. On the other hand, in 3‐ (Rowland et al., 2012) and/or 4‐year‐olds (Rowland et al., 2012; Savage et al., 2003; Shimpi et al., 2007) simply hearing a particular sentence construction results in an increased preference for using that construction. The fact that children aged 2;0–3;0 seem to be primed in experimental contexts only if they have produced the prime themselves may be related to memory and/or attention differences in comparison to older children (Shimpi et al., 2007). These studies suggest that stronger priming effects might be expected when younger children both hear and repeat the prime sentence.

### 1.4 The present study

The aim of the present study was to investigate (a) whether infinitival *to* omission and/or provision in I‐WANT‐to‐V contexts can be primed in an experimental setting by both WANT‐to and WANT‐X constructions, (b) whether priming occurs in the absence of children repeating a prime sentence, and (c) how any observed effects change with age. The broader aim was to determine how the findings might fit with the wider theoretical debate around the role of priming as implicit learning in language acquisition.

## 2 Method

### 2.1 Participants

Seventy‐five typically developing 2;6–3;0 (*M* = 2;9, *N* = 36) and 3;6–4;0 (*M* = 3;9, *N* = 39) monolingual English‐speaking children (male: 37, female: 38) from the Brighton‐and‐Hove area of the United Kingdom took part in the study. A further five children were recruited but were excluded from the study due to experimenter error (1), children not being available for the second test session (2), and children being fussy/not concentrating in the task (2).

### 2.2 Design

A 2 × 2 × 3 mixed design was used with two between‐subjects variables: Age (2;6–3;0‐year‐olds; 3;6–4;0‐year‐olds) and Priming Mode (hear‐only; hear‐and‐repeat), and one within‐subjects variable: Prime Context (WANT‐to; WANT‐X; Control) to allow for a rigorous testing of the effect of different primes on the provision of infinitival *to* within an individual child. The children were randomly allocated either to the hear‐only or hear‐and‐repeat condition.

### 2.3 Materials

Our materials section is divided into (a) Discourse contexts, (b) Elicitation targets, (c) Controlling for verb frequency, and (d) Sentence subject pronouns. We will explain these in turn below.

#### 2.3.1 Discourse contexts

##### 2.3.1.1 WANT‐to and WANT‐X contexts

Six different discourse contexts: *breakfast*,* candles*,* ball games*,* riding a bike*,* drawing*, and *clothes* were created to model WANT‐to and WANT‐X primes. These contexts consisted of two prime sentences modeling either the WANT‐to or WANT‐X construction according to prime type (e.g., WANT‐to: *I want to get my bike*; WANT‐X*: I want my big bike now*) and two nonprime sentences, that is, sentences that did not include the verb *want* (e.g., WANT‐to: *I like it very much*; WANT‐X: *I'd like to get it*). The discourse sentences were 5–6 words long. Tables 1 and 2 illustrate the prime and nonprime sentences for the WANT‐to and WANT‐X prime types for the target sentence “*I want to ride my bike*.”

**Table 1:** Examples of the test context: “Riding my bike” for hear‐only and hear‐and‐repeat WANT‐to conditions

Sentence Function	Mode	Sentence
*(a) WANT‐to context in the hear‐only condition*		
Prime	Heard	I want to get my bike
Nonprime	Heard	I like it very much
Prime	Heard	I want to ride it
Nonprime	Heard	I need my helmet as well
Complement verb given	Heard	I push my bike
Matrix clause given	Heard	I want …
Target	Elicitation	… (to) push it/bike/my bike
*(b) WANT‐to context in the hear‐and‐repeat condition*		
Prime	Heard‐and‐repeated	I want to get my bike
Nonprime	Heard‐and‐repeated	I like it very much
Prime	Heard	I want to ride it
Nonprime	Heard	I need my helmet as well
Complement verb given	Heard	I push my bike
Matrix clause given	Heard	I want …
Target	Elicitation	… (to) push it/bike/my bike

**Table 2:** Examples of the test context: “Riding my bike” for hear‐only and hear‐and‐repeat WANT‐X conditions

Sentence Function	Mode	Sentence
*(a) WANT‐X context in the hear‐only condition*		
Prime	Heard	I want my big bike now
Nonprime	Heard	I'd like to get it
Prime	Heard	I want my helmet also
Nonprime	Heard	I need to ride my bike
Complement verb given	Heard	I push my bike
Matrix clause given	Heard	I want …
Target	Elicitation	… (to) push it/bike/my bike
*(b) WANT‐X context in the hear‐and‐repeat condition*		
Prime	Heard‐and‐repeated	I want my big bike now
Nonprime	Heard‐and‐repeated	I'd like to get it
Prime	Heard	I want my helmet also
Nonprime	Heard	I need to ride my bike
Complement verb given	Heard	I push my bike
Matrix clause given	Heard	I want …
Target	Elicitation	… (to) push it/bike/my bike

To rule out the possibility that lexical priming of the word *to* could result in a higher level of infinitival *to* provision after WANT‐to primes, the nonprime sentences in the WANT‐X contexts contained an infinitival *to* construction that did not have the matrix verb *want* (e.g., *I need to ride my bike*). Conversely, the nonprime sentences in the WANT‐to discourse contexts contained those same matrix verbs as were used in the nonprime sentences in the WANT‐X contexts but in simple sentence constructions (e.g., *I need my helmet as well*). By controlling for the exposure to the verbs and infinitival *to* this way, any observed priming effects should not be related to lexical differences between prime (WANT‐to vs. WANT‐X) contexts.^4^

Controlling exposure to the target items carried with it the inclusion of nonprime sentence types which, arguably, could interfere with the intended priming effects. However, the usage‐based approach adopted here assumes that children are developing abstract grammatical representations only gradually, that it takes time for these constructions to link up. Until the link between related constructions has been made, children lack adult‐like awareness that these independent constructions share similar meanings and/or functions. This leads to conservative use, whereby initially young children rarely expand the use of a learned construction beyond the specific context(s) in which it is learned (e.g., Abbot‐Smith & Tomasello, 2006; Akhtar & Tomasello, 1997; Lieven, Pine, & Baldwin, 1997; Olguin & Tomasello, 1993; Wilson, 2003). Therefore, we can assume that the *I‐need‐to‐VP* and *I‐have‐to‐VP* constructions utilized in our study as carriers for infinitival *to* are unlikely to prime *I‐want‐to‐VP* because at the age of 2;6–4;0 these constructions are still in the process of being acquired, suggesting that children will not yet have an abstract, *I‐V‐to‐VP* construction, but instead operate with lexically specific WANT‐to and NEED‐to constructions.

See Appendix A for all sentence stimuli, targets for WANT‐to, WANT‐X, control, and filler contexts.

##### 2.3.1.2 Control contexts

To establish the baseline infinitival *to* provision rate, six control contexts (*cooking*,* towers*,* TV*,* cutting with scissors*,* going to bed*,* Buzz Lightyear*) and associated control discourse sentences were created. These discourse sentences did not contain the words *want* or *to* (e.g., *I will build a tower*). They were all 5–6 words long.

##### 2.3.1.3 Filler contexts

To keep the hear‐and‐repeat children motivated in the task (by including relatively easy discourse sentences for them to repeat), and to create a more natural discourse context in which the interaction did not solely consist of a small number of sentence types, four filler contexts (*trains*,* toys*,* cleaning*,* pushchair*) and filler discourse sentences were created, consisting of short simple sentences that did not contain the words *want* or *to* (e.g., *I do hoovering*).

Four orders for the presentation of different discourse contexts were created in which WANT‐*to* and WANT‐X target contexts alternated, interspersed with filler and control contexts (as detailed below). Within each of these four orders, two counterbalanced scripts were created, one starting with a WANT‐X context and then alternating, the other with a WANT‐*to* context and then alternating. Thus, there were eight orders of presentation in total, counterbalancing both the order of the contexts and the order of the WANT‐*to* and WANT‐X primes. Children were randomly allocated to one of these orders of presentation on Day 1, and another on Day 2. WANT‐*to* and WANT‐X (i.e., target) contexts were always separated by either a control or filler context to minimize priming effects between different discourse contexts and to create a more natural test situation with minimum repetition of one particular sentence type. As a result, the order of a given testing sequence was always the following: Target > Control > Target > Filler > Target > Control > Target > Filler > Target > Control > Target.

#### 2.3.2 Elicitation targets

##### 2.3.2.1 WANT‐to, WANT‐X, and control target sentences

Twelve target sentences were created, all of which had the structure I‐WANT‐*to*‐V(‐NP) (e.g., *I want to push my bike*). All target complement verbs were transitive (*blow*(*out*), *color*,* push*,* throw*,* wash*,* wipe*,* carry*,* cut*,* dry*,* finish*,* break*,* choose*), but we accepted intransitive uses of these (e.g., *I want* (*to*) *push*) by the children. The frequency of the complement verbs across contexts was controlled (see *Controlling for verb frequency* below for how target verbs were selected). Photographs were used to accompany target sentences and depicted multiclausal scenes of mental‐states and actions (see Appendix B).

Six target sentences were used to test the provision of *to* after discourse containing WANT‐*to* or WANT‐X primes. Each of these six sentences was elicited twice: once after a WANT‐X prime and once after a WANT‐*to* prime. The different primes (WANT‐*to*, WANT‐X) for a given target sentence (e.g., *I want to push*) were presented in different test sessions on different days with the order counterbalanced across children to avoid potential carry‐over effects; thus, children were tested on three WANT‐*to* and three WANT‐X prime contexts at each of two test sessions. The two sessions were administered between 2 and 27 days apart (*M* = 8 days, *SD *= 5.62). Although priming can sometimes carry‐over several days, this effect has only been found in older children in conditions in which the prime structure occurs with several different verbs (Savage et al., 2006). Also, by controlling children's exposure to both prime types on both testing days, the likelihood of either structure being primed across testing days more than the other was considered to be very low.

The remaining six target sentences tested the provision of *to* after control contexts (three per test session). Different sets of control (and filler) contexts and target sentences were created for the two test sessions for two reasons. First, introducing new pictures during the second session made the task more enjoyable for the child. Second, having several new “stories” (i.e., control and filler contexts) during the second session was intended to reduce the children's awareness that they had produced responses to the same WANT‐*to*/WANT‐X targets also during the first test session.

##### 2.3.2.2 Filler elicitation sentences

Four filler elicitation sentences were created, two for each test session. These were accompanied by pictures depicting scenes designed to elicit simple sentences.

#### 2.3.3 Controlling for verb frequency

Since distributional frequency patterns have been shown to affect children's language development and processing (e.g., Cameron‐Faulkner, Lieven, & Tomasello, 2003; Diessel, 2004; Kidd, Lieven, & Tomasello, 2006), we controlled for the frequency of the complement verbs that were used in infinitival *to* sentences in the WANT‐to and WANT‐X discourse contexts, and in the 12 target sentences (Appendix C lists the different complement verbs used and their frequencies). For this we extracted frequency counts from the Manchester corpus (Theakston et al., 2001). Twelve complement verbs were selected for the infinitival WANT‐to‐V prime and V‐to‐V nonprime sentences in WANT‐to and WANT‐X contexts respectively. The complement verb in the first of the two infinitival *to* sentences presented in each discourse context (e.g., Table 1, *get*) had a higher frequency than the complement verb presented in either the second infinitival sentence (e.g., Table 1, *ride*) or the target sentence (e.g., Table 1, *push*). The hear‐and‐repeat children were asked to repeat the first prime and nonprime sentence in each discourse context, so by selecting relatively high‐frequency complement verbs in these sentences, we were hoping to achieve high repetition rates. The target sentence verbs always had relatively low frequencies to minimize the likelihood of children having a prior preference to use these complement verbs in a particular (i.e., WANT‐to‐V vs. *WANT‐V) construction.

#### 2.3.4 Sentence subject pronouns

All sentences had the same sentence subject, *I*, and were accompanied by photographs depicting the Experimenter performing an action. The first person singular subject pronoun was chosen because young children are rather egocentric and the I‐WANT sentence frame is extremely frequent (approx. 40%) in children's language relative to other uses of the verb WANT (Kirjavainen & Theakston, 2011). Note, that the pronoun “I” is similarly highly frequent in WANT‐to (approx. 33%) and WANT‐X (approx. 40%) contexts, so any advantage or disadvantage of utilizing this choice of sentence subjects would be expected to influence both of our experimental conditions equally. Use of a third‐person singular subject (e.g., the lion) would require the use of a marked verb form (*wants*) which is infrequent in children's early speech. We also felt that our 2;6–3;0‐year‐olds might have struggled with actions/sentences with multiple characters (e.g., a tiger and a lion) which would license the use of the third‐person plural subject (they) and bare form of the matrix verb (i.e., *want*). It is not pragmatically ideal to ask the hear‐and‐repeat children to repeat discourse sentences with the subject *I* when the accompanying picture shows the Experimenter performing an action. However, when repeating the discourse sentences, only a handful of (our older) children changed the 1sg pronoun (*I*) into a 2sg pronoun (*you*). For the target sentences this was not a problem as the children were given the beginning of the target elicitation sentences by the experimenter and asked to complete them (E: *In this picture I want* …).

### 2.4 Procedure

The children were tested individually in a quiet room/area of their nursery, or in the case of five children in a quiet area of their home. Before the testing started, the Experimenter told the children that they were going to play a funny game on a computer and make a lovely picture with stickers. They would see some pictures on the computer screen and the Experimenter would tell them little stories about herself. The hear‐and‐repeat children were told that they would also have to pretend to be a parrot and sometimes say the same thing as the computer. All children were told that they would sometimes have to tell the Experimenter what was going on in a picture (i.e., the target elicitation).

#### 2.4.1 Warm‐up

To familiarize the children with the nature of the task and to encourage all children to engage in language production before the testing started, children took part in a warm‐up task. The warm‐up consisted of 12 sentences; six simple sentences representing three different construction types (2 × *That's X*, 2 × *There's X on the table*, 2 × *I play with X*), 2 instances of the WANT‐to construction, 2 instances of the WANT‐X construction, and 2 instances of the LIKE‐to construction. Four orders were created and children randomly assigned to them. Pre‐recorded sentences accompanied by a picture on a laptop computer screen depicting the relevant scene were played back. The first sentence (e.g., *That's a jigsaw puzzle*) of each sentence type was always repeated by the Experimenter to demonstrate what was required of the child. The second instance of that sentence type was then played back to the child and s/he was asked to repeat it (e.g., *That's a crane*). To motivate the children in the task, when the children repeated (or attempted to repeat) the sentence, they were given a sticker to put on a colorful drawing. If the child did not produce a verbatim repetition of the warm‐up sentence, the Experimenter reminded the child that he or she was pretending to be a parrot and had to say exactly the same thing as the computer. If the child did not repeat the sentence, the Experimenter played it back again and asked the child to repeat it. Once all warm‐up sentence pairs had been repeated in this manner, the Experimenter proceeded to the test items.

#### 2.4.2 Test

Children in the hear‐and‐repeat condition were asked to repeat the first two sentences (i.e., one prime and one nonprime sentence, or two simple sentences in control and filler contexts). This ensured that the children had the opportunity to repeat one instance of the matrix verb *want*, the complement verb, and infinitival *to* in both WANT‐to and WANT‐X contexts, while controlling for the possible influence of having produced sentences of any type prior to producing the target. If a hear‐and‐repeat child did not attempt to repeat a sentence, the Experimenter played that sentence back once more and asked the child to repeat it. If the child did not comply, the Experimenter moved on but asked the child to repeat the second instance of that sentence type (i.e., sentence number three) in that discourse context. This meant that for some targets, due to repetition of the sentences when children did not repeat as requested, some children could have received more input primes (3 or 4 input primes instead of 2) than others. This occurred for 13% of the TARGET SENTENCES (of these, 83% were heard WITH THREE PRIME REPETITIONS, 14% four times, and 3% five times). Kirjavainen and Theakston's (2011) study on the priming effect of WANT‐to and WANT‐X constructions found that the number of times the children had heard the prime construction did not have a significant effect on the strength of priming. As most of the TARGET sentences in our current study that received more than two INPUT PRIME repetitions were heard just three times, the input differences were ignored for the purposes of our current analysis. The hear‐only children were instructed only to listen to the sentences in the discourse context.

To make the elicitation task easier for the children and to minimize the use of nontarget complement verbs during target elicitation, in between the discourse context and target, the children were shown a picture of the Experimenter performing the action conveyed by the target complement verb (e.g., pushing) and the Experimenter explained what was going on in the picture (e.g., *In this picture*,* I **push** my bike*) and pointed at the picture. Hence, the children were given the verb and the object NP that they were expected to produce in the target sentence. They were then presented with the target picture, which depicted the Experimenter and a thought bubble. Inside the thought bubble was the same picture that the children had just seen (e.g., a picture of E pushing her bike, see Appendix B). We used a sentence completion technique (Pickering & Branigan, 1998; Rowland et al., 2012) for the target elicitation. In this, the Experimenter gave the child the matrix clause of the target sentence, for example, *And when I get tired*,* rather than riding it*,* I want* … and the child had to produce the complement clause, for example, *to push (it/bike)* versus *__ *push (it/bike)*. This procedure meant that the child only had to produce one (*push*) or two words (*to push*) for a valid response.

#### 2.4.3 Coding

During the test sessions the Experimenter wrote down children's responses online. The target sentences were coded in the following three categories.

1. Infinitival *to* provided. Target sentences in which the child produced *to* between the matrix verb and the complement verb were coded in this category. Schwa realizations of *to* (8% of 2;6–3;0‐year olds' and 6% of 3;6–4;0‐year olds' responses) were coded as attempts to produce *to* for the following reasons. First, Peters (2001) argued that before children have fully acquired the phonological and morphosyntactic properties of unstressed syllables (e.g., *to*) they tend to replace these words with schwas to preserve the number of syllables and/or the rhythm of the sentence. Second, we searched the child‐directed speech in the Manchester corpus (Theakston et al., 2001) and found that only 2.3% of realizations of the word WANT (being followed by any word) were instances of *wanna*. This means that the contracted form (i.e., *wanna*) is unlikely to be acquired early for many children learning British English (although there may be some individual differences as a function of home dialect), which in turn means that schwas in our experimental data are more likely to be attempts to provide *to* than *wanna*. Third, we used the sentence completion method in our target elicitation, that is, the children were given the beginning of the target sentence (*I want* …) by the experimenter. As *want *+ *na* is not an acceptable realization of *wanna* it is likely that if the child produced a schwa after the pre‐given WANT he or she was trying to articulate *to*.
2. Infinitival *to* omitted. Target sentences in which the child did not produce *to* between the matrix verb and the complement verb were coded in this category^5^
3. No valid response. The following three types of target responses were coded in this category:
  (a) the child did not respond
  (b) the child produced an adjective (E: *I want…* Child*: dirty*) response (*N *= 2).
  (c) the child produced a noun response (e.g., E: *I want…* Child: *bike*) (*N* = 58). Since some nouns, when produced in isolation (e.g., without determiners, demonstratives, pronouns, or adjectives), can be ambiguous as to whether they are nouns or verbs (e.g., book, plate, picture), we searched the child‐directed speech of the Manchester corpus (Theakston et al., 2001) for the bare nouns that we found in our experimental data. None were used as verbs in the corpus; thus, the children were exceedingly unlikely to have been using them as verbs with omitted infinitival *to* in the experimental context.

The sessions were audio recorded on a laptop computer using Audacity software. Any responses marked as ambiguous by the Experimenter during the test session were later checked against the audio recordings. All responses from 16 test sessions (11% of the data) were second coded by a trained coder. Agreement on whether the child produced infinitival *to*, omitted infinitival *to*, or failed to produce a response containing a verb was 81.25%, kappa = 0.613 indicating a good level of agreement. Whenever there was a discrepancy between the two coders, the Experimenter's response was retained. This was because the Experimenter coded the responses during the test sessions, and thus had both visual and auditory information available to inform their coding (the second coder had auditory information only). However, as a control, we also checked for confirmation bias in cases where there was disagreement to ensure that the Experimenter's coding was not influenced by her knowledge of the condition in which a response was produced. In these cases, the Experimenter was equally likely to code the responses as including infinitival *to*, irrespective of experimental condition (control, WANT‐to, WANT‐X, Fisher's exact *p* = .82) demonstrating that confirmation bias did not influence the coding.

To control for any output‐priming of key lexical items between WANT‐to and WANT‐X conditions, as explained above, the hear‐and‐repeat children were asked to repeat one prime (e.g., *I want my big bike now*) and one nonprime (e.g., *I like to get it*). The hear‐only children were asked to only listen to the prime and nonprime sentences. However, in practice some of the children in the hear‐only condition repeated some of the primes (and/or nonprimes), while some children in the hear‐and‐repeat condition did not always repeat a prime (and/or nonprime) sentence. Consequently, for the purposes of analysis, we coded each individual target sentence according to whether or not the children repeated at least one prime (or, for control contexts, one simple sentence) before that target regardless of which condition they were allocated to.

## 3 Results

**Figure 1:**
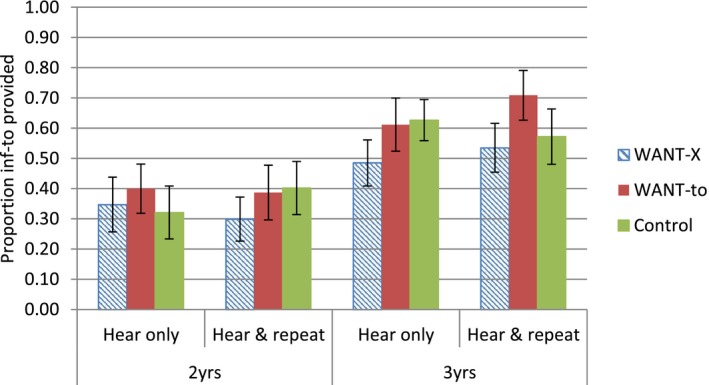
The provision of infinitival *to* in targets following WANT‐X, WANT‐to, and Control contexts (with *SE*).

The aim of the study was to test whether different WANT constructions in discourse affect the provision of infinitival *to* in subsequent WANT‐to‐V targets. For this we calculated the overall provision of infinitival *to* in targets following WANT‐to, WANT‐X, and control contexts for both hear‐only and hear‐and‐repeat conditions (defined according to the child's repetition or otherwise of the prime sentences, see above). Only responses coded as infinitival *to* omitted or infinitival *to* provided were included in the analyses (see Fig. 1).

Fig. 1 shows that, as expected, the 2‐year‐olds omit infinitival *to* in target sentences more frequently than the 3‐year‐olds. In terms of WANT‐to and WANT‐X contexts, the two age groups seem to be doing similar things. Both groups provide *to* more often after WANT‐to than WANT‐X discourse in both prime conditions (hear‐only, hear‐and‐repeat). However, the pattern of results in relation to the control condition appears less consistent, as we might expect given the changing nature of the children's linguistic representations over development, and as a result of individual differences.

To investigate the provision of infinitival *to* after WANT‐to and WANT‐X contexts, we fitted a mixed effects model to the data (Baayen, Davidson, & Bates, 2008). We wanted to establish whether producing and/or hearing WANT‐to or WANT‐X constructions results in children deviating from their relative provision of *to* in control contexts. The following variables served as fixed effects: (a) age (2 years, 3 years), (b) discourse context (WANT‐to, WANT‐X, control), (c) repetition of prime (hear‐only, hear‐and‐repeat) and (d) session (1, 2). Random effects were participant and verb (models with additional random slopes failed to converge, hence higher order terms were removed from the model). Interactions between age, discourse context, and repetition of the prime were also included in the model to establish whether the different prime types had different effects over the course of development, and whether repeating a prime has a greater effect on the provision/omission of *to*.^6^ Sum contrast coding was used for the variables age, discourse context, and repetition of prime. To test whether there was a significant three‐way interaction between these variables, we compared models with and without the three‐way interaction term. Model comparison revealed this interaction to make a significant contribution to the model fit (χ^2^(2) = 6.34, *p* = .041). To explore the interaction, separate models were fitted to the data for the two age groups. Table 3 shows the coefficients for the models for each age group.

**Table 3:** Model coefficients

	Estimate	*SE*	*z* value	*p‐*value
*Two‐year‐olds*				
(Intercept)	−1.903	0.578	−3.292	.001***
Discourse (WANT‐to vs. control)	0.355	0.176	2.018	.044*
Discourse (WANT‐X vs. control)	−0.255	0.176	−1.447	.148
Repeat prime	0.106	0.406	0.260	.795
Session2	0.581	0.252	2.307	.021*
Discourse (WANT‐to vs. control) × Repeat prime	−0.119	0.175	−0.682	.495
Discourse (WANT‐X vs. control) × Repeat prime	−0.225	0.175	−1.285	.199
*Three‐year‐olds*				
(Intercept)	−0.094	0.425	−0.221	.825
Discourse (WANT‐to vs. control)	0.638	0.184	3.471	.001***
Discourse (WANT‐X vs. control)	−0.669	0.177	−3.783	<.001***
Repeat prime	0.084	0.390	0.214	.830
Session2	1.358	0.232	5.842	<.001***
Discourse (WANT‐to vs. control) × Repeat prime	0.289	0.163	1.770	**.077 (marg)**
Discourse (WANT‐X vs. control) × Repeat prime	−0.034	0.154	−0.221	.825

*p < .05, **p < .01, ***p < .001.

Inspection of the model coefficients revealed that for both age groups, there was an effect of session, such that children produced more instances of infinitival *to* in the second than in the first session, presumably reflecting greater familiarity with the task. For the 2‐year‐olds, there was an effect of WANT‐to primes (relative to controls) such that provision of infinitival *to* increased after these primes (*M* = .39, control contexts *M* = .34), but there was no effect of WANT‐X primes (relative to controls, *M* = .32) or repetition of the prime, and no interaction between prime type and repetition of the prime. For the 3‐year‐olds, there was an effect of WANT‐to primes such that provision of infinitival *to* increased following these primes (*M* = .66) relative to controls (*M* = .60). Unlike in the younger group, for 3‐year‐olds there was also an effect of WANT‐X primes such that provision of infinitival *to* decreased following these primes (*M =* .51). The overall three‐way interaction between age, prime type and repetition of the prime is evidenced by a marginal interaction in the 3‐year data between WANT‐to primes and repetition such that the older children increased their provision of *to* after WANT‐to primes when they repeated the prime (*M *= .71), but not when they only heard the sentence (*M* = .61); a trend in the opposite direction in the 2‐year‐old data contributes to the overall interaction.^7^

## 4 Discussion

To test the suggestion that constructional competition contributes to infinitival *to* omission errors in 2‐ and 3‐year‐old children, we conducted a priming study investigating the rate of infinitival *to* omissions in obligatory contexts following different types of prime sentences. We predicted, in line with priming as implicit learning accounts, that the changing strength of the WANT‐X and WANT‐to sentence constructions in young children's linguistic repertoires would lead to differential priming effects across development, and that both hearing and repeating the prime may lead to stronger priming effects than hearing the prime only.

For the remainder of this paper, we will focus on the following: (a) the priming effect found in the two age groups, (b) the role of hearing versus hearing and producing the prime, and (c) potential additional factors contributing to infinitival *to* omissions in children.

### 4.1 The priming effect in the two age groups

We found that the 2‐year‐olds were primed by WANT‐to discourse such that they were more likely to subsequently produce infinitival *to* following WANT‐to than control contexts. In contrast, there was no effect of WANT‐X discourse in this age group in comparison to control contexts. The 3‐year‐olds were primed in a different way: first, WANT‐X primes were significantly more likely to result in omission of infinitival *to* in the subsequent targets than in control contexts. Second, WANT‐to primes were significantly more likely to result in subsequent production of infinitival *to* relative to control contexts. Thus, all children were primed, but the 2‐ and 3‐year‐olds were primed slightly differently.

We suggest that these different patterns of priming in the two age groups result from the two WANT constructions having different representational strengths at different developmental points. The input of children aged 2;6–3;0 contains more instances of the WANT‐X than WANT‐to construction, so on these grounds alone we would expect the WANT‐X construction to be learned earlier. But it is also possible that children are particularly focused on ownership and possession of objects. Although both the WANT‐X and WANT‐to constructions typically carry at least the implication of action and/or possession (e.g., *to get*,* to have*), WANT‐X places a greater focus on the object, WANT‐to on the action. One possibility is that children initially rely on the WANT‐X construction, because its meaning maps onto their interests and reflects the highly frequent contexts in which an adults' focus was placed more directly on the object than any associated action, and erroneously extend this constructions to contexts which require infinitival *to*. According to “priming as implicit learning” accounts (e.g., Bock & Griffin, 2000; Chang et al., 2006), priming effects are likely to be strongest when the sentence construction encountered fails to match the construction the child favors. Thus, if prediction plays a role in the strength of priming effects observed in the current study, then younger children's greater awareness of and preference for the WANT‐X construction could result in less violation of expectancy on hearing a WANT‐X prime, leading to the reduced priming effect we observed. In contrast, we found a facilitating effect of WANT‐to primes on the provision of *to*. This can be explained by similar mechanisms: the WANT‐to construction is less frequent in 2½–3 year‐old children's input and output than the WANT‐X construction (Kirjavainen et al., 2009), so a WANT‐to prime is likely to cause a violation of expectancy leading to stronger priming effects in this age group.

On the other hand, most children aged 3;6–4;0 have a higher level of proficiency in both the WANT‐X and the WANT‐to construction than the 2;6–3;0‐year olds, as demonstrated by our older group showing a higher rate of infinitival *to* provision in the control contexts than the younger group. Therefore, unlike the 2‐year‐olds, these older children are not likely to have a disproportionately strong representation of the WANT‐X construction relative to the WANT‐to construction, and their expectations about sentence construction following WANT may be less biased in favor of the WANT‐X construction. However, they are yet to acquire an adult‐like understanding of these form‐function mappings, as evidenced by their performance in the current study remaining considerably below ceiling. Therefore, we argue that when they encounter an instance of the WANT‐X construction in discourse leading up to the target utterance, the WANT‐X construction is available for activation and results in an increased rate of infinitival *to* omissions, whereas encountering a WANT‐to construction serves to increase subsequent provision.

Finally, at both ages our results point in the direction of children not yet having an abstract representation of the V‐to‐VP construction. If children were operating with this kind of abstract representation, we might have expected to find a priming effect for the nonprime sentences with the structure *I need to verb/I have to verb* in WANT‐X contexts, increasing provision of *to* in targets relative to control contexts. We found no such effects at either age. This result corroborates previous child language research on infinitival *to* and other complex sentence constructions. First, Kirjavainen et al. (2009) found different infinitival *to* error rates for the two most common infinitival *to* matrix verbs, WANT and *going*, in 13 children's language between the ages of 2;0 and 3;2. Different error rates indicate that children aged <3;2 are unlikely to have a fully abstract infinitival *to* construction. Second, children's utterances are initially more lexically based than those of adults, and this pattern has been observed for a range of different complex sentence types even during the fourth or fifth year of life (e.g., Brandt, Diessel, & Tomasello, 2008; Brandt, Lieven, & Tomasello, 2000, 2010; Dabrowska, Rowland, & Theakston, 2009; Diessel & Tomasello, 2001; Kidd et al., 2006; Kirjavainen et al., 2009). Priming provides researchers with one way to tap into children's underlying representations at different developmental stages, and it may be particularly useful in the case of complex sentences. Many of the verbs appearing in these structures are associated with a range of simpler constructions, too, creating a context in which competition may occur as a function of both formal and semantic overlap, reducing only as children's linguistic representations become increasingly fine‐tuned, a process that we discuss in more detail below.

### 4.2 The role of hearing versus hearing and repeating the prime

Based on previous studies (e.g., Gries, 2005; Kirjavainen & Theakston, 2011; Shimpi et al., 2007; Theakston & Lieven, 2008), we expected that the children, in particular the younger group, would show (stronger) priming if they had repeated the prime. This is not what we found. No difference was found in priming strength between the hear‐only and hear‐and‐repeat conditions in our 2;6–3;0 year old group, and in our 3;6–4;0 year old group, repeating a WANT‐to prime (but not WANT‐X prime) only marginally increased the provision of *to* in target sentences compared to when children had only heard the prime. That is, although our older group's data indicate that repeating the prime might have some effect on the strength of priming, our results overall suggest that hearing versus repeating the prime sentences do not necessarily create different priming strengths. In particular, our results demonstrate that even very young children can be primed without repeating the target constructions.

### 4.3 Additional factors contributing to infinitival to errors

It is likely that in addition to competition between two constructions, other factors also contribute to infinitival *to* errors. First, the fact that infinitival *to* holds relatively little semantic value means that children's omission of *to* does not generally result in an utterance that adults cannot comprehend; that is, the communicative effectiveness of children's erroneous infinitival *to* sentences is largely comparable to the sentences in which *to* has been produced. Even though adults do provide some negative feedback as response to their children's ungrammatical utterances (e.g., Hirsh‐Pasek, Treiman, & Schneiderman, 1984; Saxton, 2000), adults are more likely to correct children's semantically deviant utterances than utterances in which (minor) grammatical errors are produced (e.g., Brown & Hanlon, 1970). Thus, it is not unreasonable to assume that children receive little negative evidence for infinitival *to* errors. This may contribute to the fact that these errors persist in many children's speech over a number of months. Future research is needed to establish what role, if any, negative evidence plays in children's recovery from infinitival *to* omission errors.

Second, because infinitival *to* is often unstressed, it usually appears in the middle of utterances, and it is rarely uttered on its own Pinker (1984, pp. 224–227) suggested that children fail to notice *to* in adults' infinitival *to* utterances and thus make wrong predictions about their native language, namely that a null complementizer is required for to‐infinitive clauses. We agree that the lack of perceptual salience may contribute to infinitival *to* omissions early in development, but for a slightly different reason. Our usage‐based viewpoint, which emphasizes the role of language exposure in language development, assumes that if children cannot detect the to‐infinitive marker in the input consistently, they are unlikely to produce it themselves consistently. However, it is difficult to make firm predictions about how inconsistent perception might influence children's productions. For instance, if it was the case that *to* was perceptually more available with certain verbs but less so with others due to the phonological context in which it occurs, young children could initially produce *to* with some verbs but not with others. However, infinitival *to* omissions and provisions co‐occur with the same verbs (e.g., Bloom et al., 1984; Diessel, 2004; Kirjavainen et al., 2009; Landau & Thornton, 2011). On the other hand, if *to* was sometimes perceived for a given verb and sometimes not, co‐occurring errors and the provision of *to* for that verb could be expected. Establishing such a relation between perception and production would be a difficult task, and it would depend on detailed acoustic analyses of recorded data or experimental investigations (e.g., training studies) to establish more reliably what children hear, and how clear that input is to them.

Third, many matrix verbs not only take different kinds of to‐infinitive complements (e.g., *I want to hold Postman Pat*,* I want Mummy to hold Postman Pat*) as well as NP complements (e.g., *I want Postman Pat*), but they can also appear in contracted forms (e.g., I *wanna*,* gonna*,* hafta*,* needta hold Postman Pat*). Contracted forms bear similarities to both of the above constructions in that even though *wanna* takes a verbal complement it lacks a salient realization of *to* and/or could be perceived as WANT occurring with the article *a*. Although these types of contracted forms are relatively infrequent in our young British children's input and output, adult British‐English speakers readily recognize these forms. Hence, British children must learn them and their relation to other semantically and lexically similar constructions at some point in development. This variation is likely to contribute to the complexity of the task at hand in learning to‐infinitival constructions.

Fourth, it may be that children's infinitival *to* errors are to some extent caused by children combining chunks of language that they have already learned and have previously produced. Lieven, Behrens, Spears, and Tomasello (2003) found that a large proportion (63%) of one child's multiword utterances at 2 years of age were not novel, but had been produced previously over a 6‐week period. The majority (62%) of the novel utterances that the child produced could be derived from what had previously been said by simple substitutions of one item for another, for example, *Where's the bus?* for *Where's the dog*. One possibility is that children's early infinitival *to* utterances may be produced using a similar mechanism. For instance, in examples (3) and (4) the child may know the phrases *I want X* and *I need X* which she then combines with other phrases such as … *go home*, … *have a sleep* to create utterances with a clear meaning, but an ungrammatical form. Examples (5) and (6) show how this type of utterance combining might work to create ungrammatical forms of the V‐NP‐(to)‐VP construction.

3. *I want | go home.
4. *I need | have a sleep
5. *I want | Mummy hold me.
6. *I need | Daddy read story.^8^

### 4.4 Recovery from infinitival to errors

The current study along with previous child language research suggests that children acquire WANT‐to and WANT‐X constructions in their third year of life, but that these are not adult‐like even relatively late in development (e.g., Bloom et al., 1984; Kirjavainen et al., 2009; Pinker, 1984). This raises the question of when children's representations become adult‐like, and how they recover from these errors. We suggest that infinitival *to* omissions are a result of under‐specified form‐meaning mappings caused by the children being exposed to a number of similar constructions with relatively similar functions. Recovery from these errors will thus require the fine‐tuning of these form‐meaning mappings. Within the usage‐based framework adopted here, children's developing representations are best captured as the current level of abstraction derived from the input in a network of developing constructions. This means that children often have co‐occurring “correct” and “ungrammatical” forms, for example; the apparently optional marking of tense and agreement, the alternation between nominative and accusative or genitive pronouns in nominative contexts, and the inversion and noninversion of questions. Although to an adult speaker these forms are considered “ungrammatical,” this is not seen as a binary distinction in the child's grammar. In this theoretical approach acquisition is framed as a gradual tuning of form‐function mappings, such that as links between the parts of the system become more integrated, constructions that initially competed for production in a given context become more differentiated such that only the “adult” form eventually wins out. Thus, in the case of infinitival *to* omissions, it is not the case that the WANT‐X construction suddenly becomes “ungrammatical” in infinitival contexts at some point in development, but rather this is a gradual process, based on the fine‐tuning of the child's system in a probabilistic manner. By identifying the restrictions associated with the properties of the X slot, namely that it cannot permit verbs despite similarities in meaning with some NP forms, errors will cease. Note, however, that other factors will also feed into this process, as discussed above, namely the perceptual salience of *to*, the acquisition of contracted forms, and children's ever increasing knowledge of how different chunks of language can be combined together in a variety of complex sentence types.

### 4.5 Summary

In summary, the findings of the present study suggest that (a) children are acquiring at least two WANT‐constructions; (b) the representations of the WANT‐to and WANT‐X constructions compete for production; (c) immediate discourse context affects the provision/omission of *to* in subsequent utterances; (d) the priming effect of different WANT‐constructions changes across development; and (e) children as young as 2;6–3;0 years of age can show priming when they have only heard but not repeated the prime sentence. These results provide new evidence from tightly controlled contexts to support the claim that at least one factor contributing to infinitival *to* errors is competition between two (or more) related constructions (Kirjavainen et al., 2009, 2011). The results are also broadly consistent with “priming as implicit learning” accounts. Future research is needed to pin down the precise mechanisms underlying these findings, and how these effects interact with a range of other factors to contribute to infinitival *to* omission errors.

## Figures and Tables

**Figure B1:**
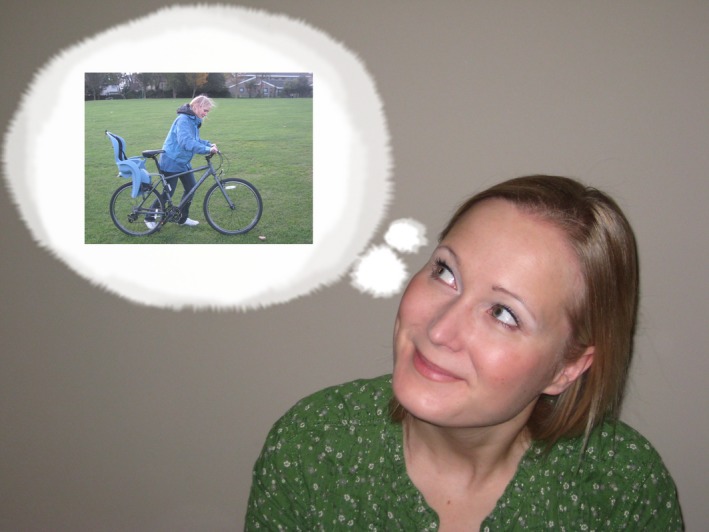
Example target sentence elicitation picture.

## Notes

1 Block capitals denote any form of that verb; small italics denote only that specific form of that verb.

2 In their analysis, Kirjavainen et al. (2009) omitted *to* from the calculation, in order to conduct a fair comparison between the number of words and morphemes produced in WANT‐to‐VP and erroneous *WANT‐VP utterances.

3 Note that although children could also be learning a *wanna* construction which competes in infinitival *to* contexts, in fact a search of the input directed to children in the Manchester corpus (Theakston et al., 2001) suggests that this is unlikely. Only 2.3% of realizations of WANT in the input were instances of *wanna*. This means that early in development, children are unlikely to have a “wanna” construction available, although there may be some individual differences dependent on the specific input received.

4 Note also that it is well documented that repetition of key lexical items between the prime and the target results in stronger priming in adults than if the prime and the target share no key lexical items (e.g., Pickering & Branigan, 1998), an effect known as *lexical boost*. However, lexical boost has been found to have little effect on the strength of priming in 3–4‐year‐old children (Rowland et al., 2012). Thus, overlap of lexical items in our age groups is unlikely to result in lexical effects.

5 We found six *wanna + *complement verb sequences in our data (2‐year olds: *N* = 1 (<1%); 3‐year‐olds *N *= 5 (1%)) when the child repeated the matrix verb uttered by the experimenter in contracted form. Due to the lack of a clearly separated schwa or infinitival *to*, these were coded as omission errors. However, given their low frequency, this does not impact on the overall results.

6 Additional models including the four‐way interaction between these variables and Session, and three‐way interactions including Session failed to converge. As Session was the least theoretically interesting variable, and inspection of the data showed that provision of infinitival *to* increase in Session 2 in all conditions, interactions with this variable were removed.

7 To check that the hear‐and‐repeat children's higher provision of *to* in target sentences after repeating WANT‐to than WANT‐X discourse stimuli does not reflect children repeating infinitival *to* in the WANT‐to discourse context more often than in the WANT‐X discourse context, we ran additional models in which the hear‐and‐repeat children who failed to repeat the nonprime sentence containing infinitival *to* (e.g., I'd like to get it) in the WANT‐X context were excluded from the analysis. The results of these models are virtually identical to the model in which all children's data are included.

8 Examples from the Manchester corpus (Theakston et al., 2001) and Thomas‐corpus (Lieven et al., 2009).

## Appendix A Sentence stimuli and targets for WANT‐to, WANT‐X, control, and filler contexts

### A.1

**Table:**

Context	Condition	Sentence Type	Sentence
Ball game	WANT‐to context	Prime (repeated in hear‐and‐repeat condition)	I want to go out
		Nonprime (repeated in hear‐and‐repeat condition)	I need my ball now
		Prime	I want to find it soon
		Nonprime	I have my little friends there
		Complement verb given	I throw the ball
		Matrix clause given	I WANT…
		Target	TO THROW (THE BALL/IT)
	WANT‐X context	Prime (repeated in hear‐and‐repeat condition)	I want my ball now
		Nonprime (repeated in hear‐and‐repeat condition)	I need to go out
		Prime	I want my little friends there
		Nonprime	I have to find the ball
		Complement verb given	I throw the ball
		Matrix clause given	I WANT…
		Target	TO THROW (THE BALL/IT)
Bike	WANT‐to context	Prime (repeated in hear‐and‐repeat condition)	I want to get my bike
		Nonprime (repeated in hear‐and‐repeat condition)	I like it very much
		Prime	I want to ride it
		Nonprime	I need my helmet as well
		Complement verb given	I push my bike
		Matrix clause given	I WANT…
		Target	TO PUSH (IT/MY BIKE)
	WANT‐X context	Prime (repeated in hear‐and‐repeat condition)	I want my big bike now
		Nonprime (repeated in hear‐and‐repeat condition)	I'd like to get it
		Prime	I want my helmet also
		Nonprime	I need to ride my bike
		Complement verb given	I push my bike
		Matrix clause given	I WANT…
		Target	TO PUSH (IT/MY BIKE)
Breakfast	WANT‐to context	Prime (repeated in hear‐and‐repeat condition)	I want to sit down
		Nonprime (repeated in hear‐and‐repeat condition)	I have my breakfast now
		Prime	I want to eat cereal
		Nonprime	I also like toast and jam
		Complement verb given	I wash my hands
		Matrix clause given	I WANT…
		Target	TO WASH (MY HANDS/THEM)
	WANT‐X context	Prime (repeated in hear‐and‐repeat condition)	I want my breakfast now
		Nonprime (repeated in hear‐and‐repeat condition)	I have to sit down
		Prime	I want lots of cereal
		Nonprime	I also like to eat toast
		Complement verb given	I was my hands
		Matrix clause given	I WANT…
		Target	TO WASH (MY HANDS/THEM)
Candles	WANT‐to context	Prime (repeated in hear‐and‐repeat condition)	I want to put candles on
		Nonprime (repeated in hear‐and‐repeat condition)	I really like big candles
		Prime	I want to look at them
		Nonprime	I have lots of them
		Complement verb given	I blow the candles up
		Target	I WANT TO BLOW (THEM) (UP)
	WANT‐X context	Prime (repeated in hear‐and‐repeat condition)	I want big candles now
		Nonprime (repeated in hear‐and‐repeat condition)	I like to put them on
		Prime	I want lots of them
		Nonprime	I have to look at them
		Complement verb given	I blow the candles up
		Matrix clause given	I WANT…
		Target	TO BLOW (THEM) (UP)
Clothes	WANT‐to context	Prime (repeated in hear‐and‐repeat condition)	I want to take clothes down
		Nonprime (repeated in hear‐and‐repeat condition)	I have lots of red tops
		Prime	I want to tip them over
		Nonprime	I need my blue trousers now
		Complement verb given	I wipe them
		Matrix clause given	I WANT…
		Target	TO WIPE (TROUSERS/THEM)
	WANT‐X context	Prime (repeated in hear‐and‐repeat condition)	I want lots of red tops
		Nonprime (repeated in hear‐and‐repeat condition)	I have to take clothes down
		Prime	I want my blue trousers now
		Nonprime	I need to tip them over
		Complement verb given	I wipe them
		Matrix clause given	I WANT…
		Target	TO WIPE (TROUSERS/THEM)
Drawing	WANT‐to context		
		Prime (repeated in hear‐and‐repeat condition)	I want to draw pictures
		Nonprime (repeated in hear‐and‐repeat condition)	I like my red pen lots
		Prime	I want to paint one
		Nonprime	I have my new crayons here
		Complement verb given	I color it
		Matrix clause given	I WANT…
		Target	TO COLOR (IT/PICTURE)
	WANT‐X context	Prime (repeated in hear‐and‐repeat condition)	I want my red pen now
		Nonprime (repeated in hear‐and‐repeat condition)	I like to draw things
		Prime	I want my crayons also
		Nonprime	I have to paint a picture
		Complement verb given	I color it
		Matrix clause given	I WANT…
		Target	TO COLOR (IT/PICTURE)
Buzz	Control	Control (repeated in hear‐and‐repeat condition)	I have Buzz Light Year
		Control (repeated in hear‐and‐repeat condition)	I like him a lot
		Control	I take him everywhere with me
		Control	I don't leave him at home
		Complement verb given	I carry it
		Matrix clause given	I WANT…
		Target	TO CARRY IT
Cooking	Control	Control (repeated in hear‐and‐repeat condition)	I think I'll make some food
		Control (repeated in hear‐and‐repeat condition)	I know I can cook
		Control	I will use some eggs
		Control	I can eat them as well
		Complement verb given	I choose a plate
		Matrix clause given	I WANT…
		Target	TO CHOOSE A PLATE
Evening	Control	Control (repeated in hear‐and‐repeat condition)	I am very tired now
		Control (repeated in hear‐and‐repeat condition)	I think I'll have a sleep
		Control	I do my teeth first
		Control	I will also comb my hair
		Complement verb given	I dry it
		Matrix clause given	I WANT…
		Target	TO DRY IT
Scissors	Control	Control (repeated in hear‐and‐repeat condition)	I have a piece of paper
		Control (repeated in hear‐and‐repeat condition)	I think it is green
		Control	I get my scissors now
		Control	I'll be very careful with them
		Complement verb given	I cut it
		Matrix clause given	I WANT…
		Target	TO CUT IT
Tower	Control	Control (repeated in hear‐and‐repeat condition)	I'll build a tower
		Control (repeated in hear‐and‐repeat condition)	I'll use my blocks
		Control	I might also get my Lego
		Control	I enjoy it very much
		Complement verb given	I break it
		Matrix clause given	I WANT…
		Target	TO BREAK IT
TV	Control	Control (repeated in hear‐and‐repeat condition)	I think I'll watch TV
		Control (repeated in hear‐and‐repeat condition)	I like Toy Story very much
		Control	I will listen carefully now
		Control	I will have a good time
		Complement verb given	I finish it
		Matrix clause given	I WANT…
		Target	TO FINISH (IT)
Trains	Filler	Filler (repeated in hear‐and‐repeat condition)	I like trains
		Filler (repeated in hear‐and‐repeat condition)	I see trains every day
		Filler	I am at the level crossing
		Filler	I get on a train
		Target	I PRESS THE BUTTON
Toys	Filler	Filler (repeated in hear‐and‐repeat condition)	I like toys
		Filler (repeated in hear‐and‐repeat condition)	I've go Eeore
		Filler	I also have Dumbo
		Filler	I stroke them
		Target	I KISS THEM/I GIVE THEM A KISS
Cleaning	Filler	Filler (repeated in hear‐and‐repeat condition)	I like cleaning my house
		Filler (repeated in hear‐and‐repeat condition)	I do the dishes every day
		Filler	I also do the dusting often
		Filler	I think it is good fun
		Target	I HOOVER/I DO HOOVERING
Pushchair	Filler	Filler (repeated in hear‐and‐repeat condition)	I have a nice push chair
		Filler (repeated in hear‐and‐repeat condition)	I take my son out
		Filler	I go down the road fast
		Filler	I can also go slowly
		Target	I PICK/LIFT HIM UP

## Appendix C The frequencies of complement verbs used in prime and target sentences

### C.1

**Table:**

	Verb	WANT‐to‐V freq	% all WANT‐to‐V	VERB‐to‐V freq	% WANT‐to‐V/VERB‐to‐V	Bare V freq	% WANT to V/Verb freq
First prime (or nonprime in WANT‐X)	Sit	74	2.68	140	53	1,505	4.92
	Go	250	9.06	766	33	8,831	2.83
	Get	142	5.15	429	33	5,622	2.53
	Take	71	2.57	214	33	1,854	3.83
	Put	215	7.80	501	43	8,556	2.51
	Draw	69	2.50	88	78	760	9.08
Second prime (or nonprime in WANT‐X)	Eat	35	1.27	155	23	1,429	2.45
	Find	18	0.65	69	26	1,647	1.09
	Ride	2	0.07	9	22	80	2.50
	Tip	12	0.44	21	57	154	7.79
	Look	76	2.76	156	49	6,220	1.22
	Paint	12	0.44	14	86	80	15.00
Target verbs for WANT‐to/X contexts	Wash	6	0.22	28	21	206	2.91
	Throw	6	0.22	21	29	445	1.35
	Push	2	0.07	18	11	350	0.57
	Wipe	6	0.22	14	43	185	3.24
	Blow	4	0.15	19	21	143	2.80
	Color	11	0.40	12	92	188	5.85
Target verbs for control contexts	Carry	5	0.18	8	63	131	3.82
	Choose	6	0.22	14	43	118	5.08
	Dry	2	0.07	10	20	72	2.78
	Cut	3	0.11	11	27	237	1.27
	Break	10	0.36	15	67	336	2.98
	Finish	12	0.44	19	63	139	8.63
